# Karyotype, C-banding and AgNORs of two endemic leuciscine fish, *Pseudophoxinus crassus* (Ladiges, 1960) and *P. hittitorum* Freyhof & Özulug, 2010 (Teleostei, Cyprinidae)

**DOI:** 10.3897/CompCytogen.v8i4.7623

**Published:** 2014-10-31

**Authors:** Sevgi Unal, Muhammet Gaffaroğlu, Muradiye Karasu Ayata, Eşref Yüksel

**Affiliations:** 1 Gazi University, Science Faculty, Department of Biology, Ankara, Turkey; 2 Ahi Evran University, Science and Art Faculty, Department of Biology, Kırşehir, Turkey

**Keywords:** Karyotype, C-banding, NOR-phenotype, Leuciscinae, cytotaxonomy

## Abstract

The genus *Pseudophoxinus* Bleeker, 1860 is found in a wide range of habitats in central Anatolia, but it is not well known from a cytogenetic aspect. In this study the first karyotypic description of the spring minnows *Pseudophoxinus crassus* (Ladiges, 1960) and *Pseudophoxinus hittitorum* Freyhof & Özulug, 2010 by means of conventional methods (Giemsa staining, C-banding, silver nitrate impregnation (Ag-NORs)) was performed. Both species are endemic and have restricted distributions in Central Anatolia. *Pseudophoxinus crassus* and *Pseudophoxinus hittitorum* have the same diploid chromosome number, 2n = 50, patterns of distribution of constitutive heterochromatin (CH), and localization of nucleolus organizer regions (NORs), but differ in their karyotypic formulae (KFs). The C-banding technique revealed clear pericentromeric blocks of CH in many chromosomes; Ag-NORs treatment revealed consistent positive signals at the end of the short arms of a submetacentric chromosome pair, likely homologous in both species. The karyotypic differences found between these species can be used for their taxonomical study.

## Introduction

Spring minnows of the cyprinid genus *Pseudophoxinus* Bleeker, 1860 are distributed from Central Anatolia east to Azerbaijan and South to Israel (Freyhof and Özuluğ 2010). The genus belongs to the subfamily Leuciscinae, the major element of the Anatolia cyprinid fauna. Leuciscinae fishes include 54 species belonging to 17 genera in Anatolia, of which 26 species and subspecies are endemic. With 19 species recognized in Turkey, *Pseudophoxinus* is one of the most species-rich genera with a great number of the endemic species (Bogutskaya 1997, Freyhof and Özuluğ 2006, Bogutskaya et al. 2007, Karasu et al. 2011, Küçük et al. 2012, Küçük and Güçlü 2014). Species of this genus are found in a wide range of habitats in central Anatolia (Hrbek et al. 2004). According to IUCN, a significant point about the herein studied species is the fact that *Pseudophoxinus crassus* and *Pseudophoxinus hittitorum* are endangered (EN) species and their population trends are decreasing (IUCN 2014a; IUCN 2014b).

Karyotypic data for the genus are available only for *Pseudophoxinus antalyae* Bogutskaya, 1992 and *Pseudophoxinus firati* Bogutskaya, Küçük & Atalay, 2007 (Table 1). In both species a karyotype with 2n = 50 was revealed, indicating a conserved karyotypic evolution in relation to the diploid number (Ergene et al. 2010, Karasu et al. 2011). Thus, cytogenetic data for *Pseudophoxinus* are insufficient, and further study is needed to evaluate karyological characteristics of the genus, to improve the taxonomic identification of these fish, and to understand the evolutionary trends in this taxon (Yüksel and Gülkaç 1992).

**Table 1.** Cytogenetic data available for the genus *Pseudophoxinus*.

Species	Locality	2n	Karyotypic formula	FN	NOR	C-band	Reference
*Pseudophoxinus antalyae*	Berdan River	50	16M+14SM+12ST+8A	92	1 pair *st. p* terminal	several	Ergene et al. 2010
*Pseudophoxinus firati*	Tohma Creek	50	38M-SM+12ST	88	2 pairs *sm-st. p* terminal	6 pairs	Karasu et. al. 2011
*Pseudophoxinus crassus*	İnsuyu Spring	50	12M+30SM+8ST-A	92	1 pair *sm p* terminal	several	Present study
*Pseudophoxinus hittitorum*	Beyşehir Spring	50	14M+26SM+10ST-A	90	1 pair *sm p* terminal	several	Present study

2n: diploid number; FN: fundamental number; NOR: nucleolus organizer regions type; M: metacentric; SM: submetacentric; ST: subtelocentric; A: acrocentric; *p* short arm.

The aim of this study is to describe the karyotypes of *Pseudophoxinus crassus* and *Pseudophoxinus hittitorum*, including identification of CH blocks and NORs by conventional cytogenetic techniques (Giemsa staining, C-banding, and Ag impregnation).

## Material and methods

Specimens were captured by electrofishing in two distinct localities during the summer-autumn, 2012 and spring-summer, 2013. Three males and two females of *Pseudophoxinus crassus* were collected in Cihanbeyli-İnsuyu spring (38°42'N, 32°45'E) and four females and four males of *Pseudophoxinus hittitorum* in Beyşehir-Eflatunpınarı spring (37°52'N, 31°34'E). Specimens were transported alive to the laboratory and kept in well-aerated aquaria until analysis was performed. Chromosome spreads were obtained using standard kidney protocol (Collares-Pereira 1992). Chromosomes were stained with 4% Giemsa solution (pH = 6.8). C-bands were obtained according to Sumner technique (Sumner 1972). Silver impregnation to detect NORs followed the method of Howell and Black (1980).

The chromosome slides were observed by 100× objective with immersion oil and photographed using a Leica DM 3000 research microscope. AKAS software was used to take pictures of the metaphase figs. Measurements of chromosomes were performed by digital caliper from each individual and karyotypes were prepared manually. Chromosomes were arranged in decreasing size order and classified according to their arm ratios (Levan et al. 1964) in three categories: metacentric (M), submetacentric (SM) and subtelocentric to acrocentric (ST-A). To determine the fundamental number (FN), M and SM chromosomes were considered as bi-armed whereas those of group ST/A as uni-armed.

## Results

**Figure 1.**
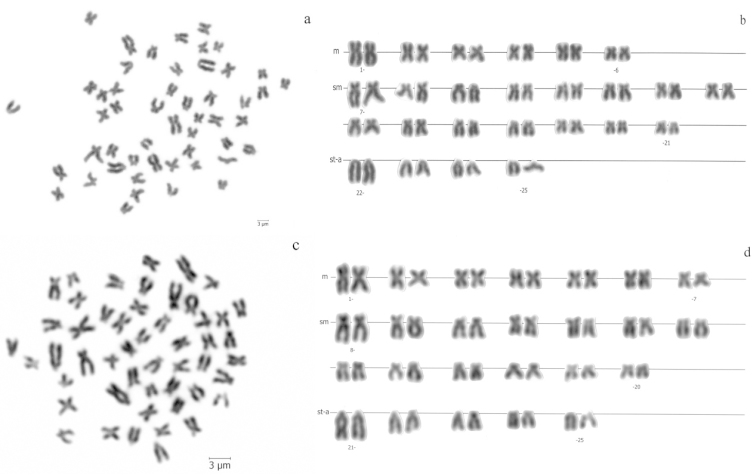
**a** Giemsa stained metaphase and **b** corresponding karyotype of *Pseudophoxinus crassus* from Cihanbeyli stream **c** Giemsa stained metaphase and **d** karyotype of *Pseudophoxinus hittitorum* from Beyşehir drainage. Scale bar = 3 µm.

243 metaphase figs were examined for *Pseudophoxinus crassus* and 266 metaphase figs – for *Pseudophoxinus hittitorum*. For *Pseudophoxinus crassus* the percentage of the finding of 50 chromosomes was 81.50%. Other percentages were: for 49 chromosomes – 14.45%, for 48 chromosomes – 2.70%, for 47 chromosomes – 1.35%. For *Pseudophoxinus hittitorum* the percentage of the finding of 50 chromosomes was 80.00%. Other percentages were: for 49 chromosomes – 13.50%, for 48 chromosomes – 3.00%, for 47 chromosomes – 2.30% and for 46 chromosomes – 1.20%. Therefore it was considered that the analyzed individuals of *Pseudophoxinus crassus* and *Pseudophoxinus hittitorum* had the same diploid numbers 2n = 50, but differed in their karyotypic formulas (KFs), which were 12 M + 30 SM + 8 ST-A (FN = 92) for *Pseudophoxinus crassus* and 14 M + 26 SM + 10 ST-A (FN = 90) for *Pseudophoxinus hittitorum*, respectively (Fig. 1). No sex chromosomes were identified for either species.

**Figure 2.**
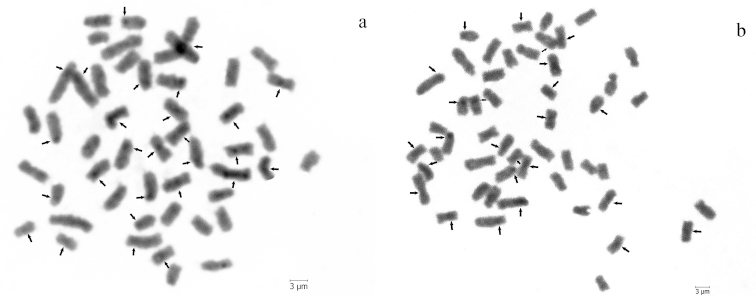
Metaphase spreads of (**a**) *Pseudophoxinus crassus* and (**b**) *Pseudophoxinus hittitorum* with C-banding. Arrows show CH regions. Scale bar = 3 µm.

C-banding revealed the presence of the blocks of constitutive heterochromatin at the pericentromeric regions of many chromosome pairs in both species (Fig. 2).

**Figure 3.**
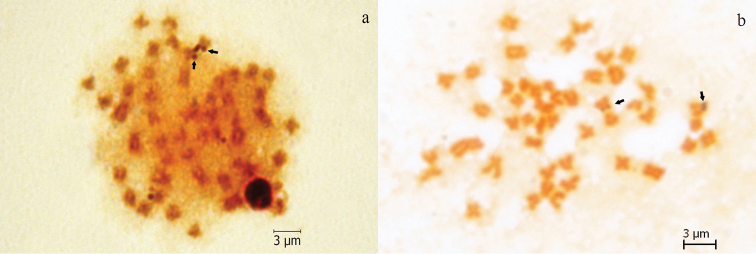
Metaphase spreads of (**a**) *Pseudophoxinus crassus* and (**b**) *Pseudophoxinus hittitorum* with Ag-NOR treatmets. Arrows show NORs. Scale bar = 3 µm.

The NORs were localized near to the secondary constriction on the short arm of a SM chromosome pair in both species (Fig. 3).

## Discussion

*Pseudophoxinus crassus* and *Pseudophoxinus hittitorum* karyotypes demonstrated the general pattern described for most Leuciscinae that have the chromosome number (2n = 50), but their KFs differed. This is consistent with most other species of the genus *Pseudophoxinus*, which share 2n = 50 and differ in their KFs (Ergene et al. 2010, Karasu et al. 2011). The chromosome sets of leuciscine cyprinids are characterized mainly by bi-armed (meta- and submetacentric) compared to the uni-armed (subtelo- and acrocentric) elements as observed in *Pseudophoxinus crassus* and *Pseudophoxinus hittitorum*. A large subtelocentric/acrocentric chromosome pair is considered as a cytotaxonomic marker for the subfamily Leuciscinae (Rab and Collares-Pereira 1995, Rab et al. 2008) and it is also present in both analysed species. However, cyprinid sex chromosomes appear to have remained morphologically undifferentiated (Sola and Gornung 2001). *Pseudophoxinus crassus* and *Pseudophoxinus hittitorum* also display the cyprinid characteristics mentioned above.

C-bands identify regions of constitutive heterochromatin, which contain transcriptionally inactive highly repetitive DNA sequences (Gold et al. 1990). The difference in heterochromatin localization can be used as cytogenetic marker for the differentiation of species and for the reconstruction of chromosome evolution in the taxa (Gaffaroğlu and Yüksel 2009). In *Pseudophoxinus crassus* and *Pseudophoxinus hittitorum* C-positive blocks were pericentromeric, as in the *Pseudophoxinus antalyae* (Ergene et al. 2010) and *Pseudophoxinus firati* (Karasu et al. 2011). It was shown, that other studied Leuciscinae species as *Acanthobrama marmid* Heckel, 1843 (Gaffaroğlu and Yüksel 2009), *Squalius anatolicus* (Bogutskaya, 1997) (Ünal 2011) and *Squalius lucumonis* (Bianco, 1983) (Rossi et al. 2012) also have CH blocks on the pericentromeric regions. This pattern is conserved in Neotelostei as a whole, and also in all the Leuciscine genera examined to date (Collares-Pereira and Rab 1999, Boron et al. 2009, Rossi et al. 2012).

The number and location of NORs have been used as chromosome markers in fish cytotaxonomy (Pereira et al. 2012, Rossi et al. 2012, Nabais et al. 2013). The NORs located on a medium-sized SM chromosome pair corresponds to those observed in many of the leuciscines analyzed (Bianco et al. 2004). In spite of the many exceptions reported in Leuciscinae species from both Eurasia and North America (Pereira et al. 2009, Rossi et al. 2012), a single pair of NOR-carrying chromosome is considered as an ancestral character in this lineage (Rab and Collares-Pereira 1995, Rab et al. 2007). Within the genus *Pseudophoxinus*, a single NOR-bearing chromosome pair as in *Pseudophoxinus crassus* and *Pseudophoxinus hittitorum*, ﻿was observed in *Pseudophoxinus antalyae* (Ergene et al. 2010) whereas multiple NOR-carrying chromosomes were detected in *Pseudophoxinus firati* (Karasu et al. 2011). Although NORs are usually located on the short arms of chromosomes, sometimes they can be seen on the long arms of metacentric and acrocentric chromosomes (Rab and Collares-Pereira 1995, Rab et al. 1996). Furthermore, NORs can be seen between telomeres and centromeres (Amemiya and Gold 1988). Generally, the NOR-phenotype is observed at the terminal on short arms of mid-sized A-ST chromosomes (Takai and Ojima 1992), and rarely at the terminal on short arms of mid-sized SM chromosomes (Gold et al. 1988, Magtoon and Arai 1993) like in *Pseudophoxinus crassus* and *Pseudophoxinus hittitorum*. Conversely to what was reported for some others leuciscin cyprinids (Ünal 2011), no NOR polymorphism was observed in the specimens from our study. Further, there is no report of any variation in NORs’ phenotype in all analyzed individuals of the genus *Pseudophoxinus* (Ergene et al. 2010, Karasu et al. 2011). Thus the karyotypes of these species conserved plesiomorphic condition that is confirmed by present study.

In conclusion, the karyotypic differences and CH and NOR localizations found in the two *Pseudophoxinus* species studied herein can be used as a cytogenetic comparison data.

## References

[B1] Amemiya CT Gold JR (1988) Chromosomal NORs as taxonomic and systematic characters in North American cyprinid fishes. Genetica 76(2): 81–90. doi: 10.1007/BF00058806

[B2] Bianco PG Aprea G Balletto E Capriglione T Fulgione D Odierna DG (2004) The karyology of the cyprinid genera *Scardinius* and *Rutilus* in southern Europe. Ichthyological Research 51(3): 274–278. doi: 10.1007/s10228-004-0221-y

[B3] Bogutskaya NG (1997) Contribution to the knowledge of leuciscine fishes of Asia Minor. Part 2. An annotated check-list of leuciscine fishes (Leuciscinae, Cyprinidae) of Turkey with descriptions of a new species and two new subspecies. Mitteilungen aus dem Hamburgischen Zoologischen Museum und Institut 94: 161–186 http://www.fishbase.org/references/FBRefSummary.php?id=33550&speccode=268

[B4] Bogutskaya NG Küçük F Atalay MA (2007) A description of three new species of the genus *Pseudophoxinus* from Turkey (Teleostei: Cyprinidae: Leuciscinae). Zoosystematica Rossica 15(2): 335–341 http://elibrary.ru/item.asp?id=11803973

[B5] Boron A Porycka K Ito D Abe S Kirtiklis L (2009) Comparative molecular cytogenetic analysis of three *Leuciscus* species (Pisces, Cyprinidae) using chromosome banding and FISH with rDNA. Genetica 135(2): 199–207. doi: 10.1007/s10709-008-9269-3 18473124 10.1007/s10709-008-9269-3

[B6] Collares-Pereira MJ (1992) *in vivo* Direct Chromosome Preparation (Air Drying Technique). 1st International Workshop on Fish Cytogenetic Techniques France, September 14–24, 1990, 15–9.

[B7] Collares-Pereira MJ Rab P (1999) NOR polymorphism in the Iberian species *Chondrostoma lusitanicum* (Pisces: Cyprinidae) reexamination by FISH. Genetica 105(3): 301–303. doi: 10.1023/A:1003885922023 10761113 10.1023/a:1003885922023

[B8] Ergene S Karahan A Kuru M (2010) Cytogenetic Analysis of *Pseudophoxinus antalyae* Bogutskaya, 1992 (Pisces: Cyprinidae) from the Eastern Mediterranean River Basin, Turkey. Turkish Journal of Zoology 34(1): 111–117. doi: 10.3906/zoo-0807-33

[B9] Freyhof J Özuluğ M (2006) *Pseudophoxinus ninae*, a new species from Central Anatolia, Turkey (Teleostei: Cyprinidae). Ichthyological Exploration of Freshwaters 17(3): 255–259 http://www.pfeil-verlag.de/04biol/pdf/ief17_3_07.pdf

[B10] Freyhof J Özuluğ M (2010) *Pseudophoxinus hittitorum*, a new species of spring minnow from Central Anatolia (Teleostei: Cyprinidae). Ichthyological Exploration of Freshwaters 21(3): 239–245 http://www.pfeil-verlag.de/04biol/pdf/ief21_3_06.pdf

[B11] Gaffaroğlu M Yüksel E (2009) Constitutive heterochromatin in *Acanthobrama marmid* and *Cyprinion macrostomus* (Osteichthyes, Cyprinidae). Kafkas Üniversitesi Veteriner Fakültesi Dergisi 15(2): 169–172 http://vetdergi.kafkas.edu.tr/extdocs/2009_2/169_172.pdf

[B12] Gold JR Zoch PK Amemiya CT (1988) Cytogenetic studies in North American minnows (Cyprinidae). XIV. Chromosomal NOR phenotypes of eight species from the genus Notropis. Cytobios 54: 137–147. doi: 10.1139/z91-398

[B13] Gold JR Li YC Shipley NS Powers PK (1990) Improved methods for working with fish chromosomes with a review of metaphase chromosome banding. Journal of Fish Biology 37(4): 563–575. doi: 10.1111/j.1095-8649.1990.tb05889.x

[B14] Gromicho M Ozouf-Costaz C Collares-Pereira MJ (2005) Lack of correspondence between CMA_3_-, Ag-positive signals and 28S rDNA loci in two Iberian minnows (Teleostei, Cyprinidae) evidenced by sequential banding. Cytogenetic and Genome Research 109: 507–511. doi: 10.1159/000084211 15905646 10.1159/000084211

[B15] Howell WM Black DA (1980) Controlled silver staining of nucleolus organizer regions with a protective colloidal developer: a 1-step method. Experientia 36(8): 1014–1015. doi: 10.1007/BF01953855 6160049 10.1007/BF01953855

[B16] Hrbek T Stölting KN Bardakcı F Küçük F Wildekamp RH Meyera A (2004) Plate tectonics and biogeographical patterns of the *Pseudophoxinus* (Pisces: Cypriniformes) species complex of central Anatolia, Turkey. Molecular Phylogenetics and Evolution 32(1): 297–308. doi: 10.1016/j.ympev.2003.12.017 15186815 10.1016/j.ympev.2003.12.017

[B17] IUCN (2014a) *Pseudophoxinus crassus*. http://www.iucnredlist.org/details/60751/0 [accessed 10 April 2014]

[B18] IUCN (2014b) *Pseudophoxinus hittitorum*. http://www.iucnredlist.org/details/19449272/0 [accessed 10 April 2014]

[B19] Karasu M Yüksel E Gaffaroğlu M (2011) Karyotype, NORs, and C-banding analysis of *Pseudophoxinus firati* Bogutskaya, Küçük & Atalay, 2007 (Actinopterygii, Cyprinidae) in the Euphrates River, Turkey. Turkish Journal of Zoology 35(6): 865–868. doi: 10.3906/zoo-0912-129

[B20] Küçük F Atalay MA Güçlü SS Gülle İ (2012) Türkiye’de Yayılış Gösteren *Pseudophoxinus* (Teleostei:Cyprinidae) Türlerinin Bazı Morfolojik Özellikleri ve Zoocoğrafik Dağılımları. Eğirdir Su Ürünleri Fakültesi Dergisi 8(2): 1–9 http://sdu.edu.tr/edergi/index.php/esufd/article/viewFile/3827/3473

[B21] Küçük F Güçlü SS (2014) A new *Pseudophoxinus* (Teleostei, Cyprinidae) species from Asi River Drainage (Turkey). ZooKeys 411: 57–66. doi: 10.3897/zookeys.411.6833 24899855 10.3897/zookeys.411.6833 PMC4042818

[B22] Levan A Fredga K Sandberg AA (1964) Nomenclature for centromeric position on chromosomes. Hereditas 52(2): 201–220. doi: 10.1111/j.1601-5223.1964.tb01953.x

[B23] Magtoon W Arai R (1993) Karyotypes and distribution of nucleolus organizer regions in cyprinid fishes from Thailand. Japanese Journal of Ichthyology 40(1): 77–85 http://www.wdc-jp.biz/pdf_store/isj/publication/pdf/40/401/40111.pdf

[B24] Nabais C Rampin M Collares-Pereira MJ (2013) Comparative cytogenetics of two endangered leuciscine fish, *Squalius aradensis* and *S. torgalensis* (Teleostei, Cyprinidae), from the Iberian Peninsula. Comparative Cytogenetics 7(1): 33–42. doi: 10.3897/CompCytogen.v7i1.4672 24260688 10.3897/CompCytogen.v7i1.4672 PMC3833748

[B25] Pereira C Neto A Collares-Pereira MJ (2009) Cytogenetic survey of species of two distinct genera of Iberian nases (Cyprinidae, Leuciscinae) that hybridize extensively in nature. I. Evidence of similar and conserved chromosome pattern with some few species-speciﬁc markers at macro-structural level. Genetica 137(3): 285–291. doi: 10.1007/s10709-009-9379-6 19585245 10.1007/s10709-009-9379-6

[B26] Pereira CS Rab P Collares-Pereira MJ (2012) Chromosomes of European cyprinid fishes: comparative cytogenetics and chromosomal characteristics of ribosomal DNAs in nine Iberian chondrostomine species (Leuciscinae). Genetica 140(10–12): 485–495. doi: 10.1007/s10709-013-9697-6 23329299 10.1007/s10709-013-9697-6

[B27] Rab P Collares-Pereira MJ (1995) Chromosomes of European Cyprinid fishes (Cyprinidae, Cypriniformes): A review. Folia Zoological 44(3): 193–214 http://serials.unibo.it/cgi-ser/start/en/spogli/dfs.tcl?prog_art=3256190&language=ENGLISH&view=articoli

[B28] Rab P Karakousis Y Rabova M (1996) Karyotype, NOR phenotype and C-banding study of *Barbus cyclolepis* from Greece. Folia Zoologica 45: 77–83 http://eurekamag.com/research/038/362/038362965.php#close

[B29] Rab P Bohlen J Rabova M Flajshans M Kalous L (2007) Cytogenetic as a tool in ﬁsh conservation: the present situation in Europe. In: Pisano E Ozouf-Costaz C Foresti F Kappor BG Enﬁeld NH (Eds) Fish cytogenetics. 2.1 Science Publisher Inc., USA, 215–240.

[B30] Rab P Rabova M Pereira CS Collares-Pereira MJ Pelikanova S (2008) Chromosome studies of European cyprinid ﬁshes: interspeciﬁc homology of leuciscine cytotaxonomic marker the largest subtelocentric chromosome pair as revealed by crossspecies painting. Chromosome Research 16(6): 863–873. doi: 10.1007/s10709-013-9697-6 18709543 10.1007/s10577-008-1245-3

[B31] Rossi AR Milana V Hett AK Tancioni L (2012) Molecular cytogenetic analysis of the Appenine endemic cyprinid fish *Squalius lucumonis* and three other Italian leuciscines using chromosome banding and FISH with rDNA probes. Genetica 140(10–12): 469–476. doi: 10.1007/s10709-012-9695-0 23238894 10.1007/s10709-012-9695-0

[B32] Sola L Gornung E (2001) Classical and molecular cytogenetics of the zebrafish, *Danio rerio* (Cyprinidae, Cypriniformes): an overview. Genetica 111(1–3): 397–412. doi: 10.1023/A:1013776323077 11841183 10.1023/a:1013776323077

[B33] Souza ACP Nagamachi CY Milhomem SSR Feldberg E Pieczarka JC (2009) Cytogenetics analysis in catfish species of the genus *Peckoltia* Miranda Ribeiro, 1912 (Teleostei: Siluriformes: Loricariidae). Comparative Cytogenetics 3(2): 103–109. doi: 10.3897/compcytogen.v3i2.17

[B34] Sumner AT (1972) A simple technique for demonstrating centromeric heterochromatin. Experimental Cell Research 75(1): 304–6. doi: 10.1016/0014-4827(72)90558-7 4117921 10.1016/0014-4827(72)90558-7

[B35] Takai A Ojima Y (1992) Chromosomal distribution of nucleolus organizer regions in Japanese Cyprinid fish. Cytobios 71(284): 7–17 http://eurekamag.com/research/002/322/002322876.php

[B36] Ünal S (2011) *Squalius anatolicus* (Bogutskaya, 1997) (Pisces, Cyprinidae)’un Sitogenetik Analizi. MSc Dissertation Science Institute, Ahi Evran University, Kırşehir, Turkey, 61 pp [in Turkish]

[B37] Yüksel E Gülkaç MD (1992) On the karyotypes in some populations of the subterranean mole rats in the lower Euphrates-basin, Turkey. Caryologia 45(2): 175–190. doi: 10.1080/00087114.1992.10797221

